# Kinetics of SARS-CoV-2 neutralizing antibodies in Omicron breakthrough cases with inactivated vaccination: Role in inferring the history and duration of infection

**DOI:** 10.3389/fimmu.2023.1083523

**Published:** 2023-01-24

**Authors:** Aidibai Simayi, Chuchu Li, Cong Chen, Yin Wang, Chen Dong, Hua Tian, Xiaoxiao Kong, Lu Zhou, Jiefu Peng, Shihan Zhang, Fengcai Zhu, Jianli Hu, Ke Xu, Hui Jin, Huafeng Fan, Changjun Bao, Liguo Zhu

**Affiliations:** ^1^ Department of Epidemiology and Health Statistics, School of Public Health, Southeast University, Nanjing, China; ^2^ Key Laboratory of Environmental Medicine Engineering, Ministry of Education, School of Public Health, Southeast University, Nanjing, China; ^3^ Department of Acute Infectious Disease Control and Prevention, Jiangsu Provincial Center for Disease Control and Prevention, Nanjing, China; ^4^ Department of Acute Infectious Disease Control and Prevention, Changzhou Center for Disease Control and Prevention, Changzhou, China; ^5^ Department of Acute Infectious Disease Control and Prevention, Yangzhou Center for Disease Control and Prevention, Yangzhou, China; ^6^ National Health Commission (NHC) Key Laboratory of Enteric Pathogenic Microbiology, Jiangsu Provincial Center for Disease Control and Prevention, Nanjing, China; ^7^ Key Laboratory of Infectious Diseases, School of Public Health, Nanjing Medical University, Nanjing, China; ^8^ Department of Microbiological Laboratory, Nanjing Municipal Center for Disease Control and Prevention, Nanjing, China; ^9^ Jiangsu Province Engineering Research Center of Health Emergency, Jiangsu Provincial Center for Disease Control and Prevention, Nanjing, China; ^10^ Jiangsu Key Lab of Cancer Biomarkers, Prevention and Treatment, Jiangsu Collaborative Innovation Center for Cancer Medicine, Nanjing Medical University, Nanjing, China

**Keywords:** SARS-CoV-2, Omicron variant, inactive CoronaVac, breakthrough infection, neutralizing antibody

## Abstract

**Background:**

The quantitative level and kinetics of neutralizing antibodies (NAbs) in individuals with Omicron breakthrough infections may differ from those of vaccinated individuals without infection. Therefore, we aimed to evaluate the difference in NAb levels to distinguish the breakthrough cases from the post-immunized population to identify early infected person in an outbreak epidemic when nasal and/or pharyngeal swab nucleic acid real-time PCR results were negative.

**Methods:**

We collected 1077 serum samples from 877 individuals, including 189 with Omicron BA.2 breakthrough infection and 688 post-immunized participants. NAb titers were detected using the surrogate virus neutralization test, and were log(2)-transformed to normalize prior to analysis using Student’s unpaired t-tests. Geometric mean titers (GMT) were calculated with 95% confidence intervals (CI). Linear regression models were used to identify factors associated with NAb levels. We further conducted ROC curve analysis to evaluate the NAbs’ ability to identify breakthrough infected individuals in the vaccinated population.

**Results:**

The breakthrough infection group had a consistently higher NAb levels than the post-immunized group according to time since the last vaccination. NAb titers in the breakthrough infection group were 6.4-fold higher than those in the post-immunized group (GMT: 40.72 AU/mL and 6.38 AU/mL, respectively; *p*<0.0001). In the breakthrough infection group, the NAbs in the convalescent phase were 10.9-fold higher than in the acute phase (GMT: 200.48 AU/mL and 18.46 AU/mL, respectively; p<0.0001). In addition, the time since infection, booster vaccination, and the time since last vaccination were associated with log(2)-transformed NAb levels in the breakthrough infection group. ROC curve analysis showed that ROC area was largest (0.728) when the cut-off value of log(2)-transformed NAb was 6, which indicated that NAb levels could identify breakthrough infected individuals in the vaccinated population.

**Conclusion:**

Our study demonstrates that the NAb titers of Omicron BA.2 variant breakthrough cases are higher than in the post-immunized group. The difference in NAb levels could be used to identify cases of breakthrough infection from the post-immunized population in an outbreak epidemic.

## Introduction

Up to December 2, 2022, more than 639 million confirmed cases of coronavirus disease 2019 (COVID-19) were reported worldwide, with a total of more than 6.6 million deaths (https://covid19.who.int). Although several vaccines have been approved and numerous people have been vaccinated worldwide, the pandemic has not yet been effectively controlled. Breakthrough SARS-CoV-2 infections are defined as infections that occur at least 2 weeks after an individual has been fully vaccinated (1–3).

The B.1.1.529 SARS-CoV-2 variant was first identified in South Africa on November 9, 2021, and was defined by WHO as a new variant of concern, Omicron, on November 26, 2021, because of the sharp increase in the number of cases (4, 5). Previous studies have found that plasma from convalescent patients or individuals vaccinated with two doses of the different COVID-19 vaccines show very low levels of neutralizing antibodies (NAbs) or no neutralizing activity compared with individuals infected with the Omicron variant (6–8). NAbs are responsible for defending cells from pathogens, and are effective at neutralizing pathogens, reducing viral load, and protecting tissues or cells from infection, thus providing immunity. Production of these antibodies, which are stored in the body for a longer period than therapeutic antibodies, may be increased by vaccination or by previous infection. NAbs are used to treat infections, predict disease outcomes (9–11) and assess of vaccine efficacy and the ability to prevent reinfection, such as Enterovirus 71 (12, 13). However, the role of NAb quantification in inferring the history and time since infection has not been well studied.

Owing to the dual influence of infection and vaccine, the quantitative level and kinetic characteristics of NAbs in individuals with breakthrough infections with the Omicron variant may differ from those of individuals immunized with inactivated vaccines only, without infection. In the management of a COVID-19 aggregated outbreak, identifying early infections, and tracing the source of the epidemic/clusters of COVID-19 patients play an important role in controlling the source of infection, reducing the spread of the outbreak, and ultimately controlling the outbreak. The main method to determine infection is to detect of viral nucleic acid, which often has a short shedding period of 1-2 weeks. However, a large proportion of SARS-CoV-2 infected patients are asymptomatic, suspected early infected patients are often already negative when tested for nucleic acid, while the half-life of antibodies often exceeds 1 month, and antibody titers also show dynamic changes with the duration of infection. If differences in NAbs between breakthrough cases and post-immunized populations can be confirmed, this property can be exploited to use NAbs as an immunological indicator to aid in the diagnosis of early infected person in an outbreak epidemic. This prompted us to conduct a case-control study to establish a quantitative association to predict the duration of infection, which could provide additional scientific and technical tools for tracing the source of the epidemic/clusters of COVID-19 patients when nasal and/or pharyngeal swab nucleic acid real-time PCR results were negative.

We first examined the neutralizing response of individuals with breakthrough infections caused by the Omicron variant in an outbreak Jiangsu Province, China, from February to May, 2022 and compared it with those of vaccinated individuals without infection. We then analyzed the difference in NAb levels between the acute and convalescent phases of participants with breakthrough infections. Finally, we established a regression equation to illustrate the factors influencing NAb levels in participants with breakthrough infections, including the time since infection. These results contribute to expanding our ability to distinguish the quantitative level of NAbs induced by vaccination alone, and by breakthrough infection, and to estimate the time since infection among individuals with breakthrough infection during the large epidemic of the Omicron variant.

## Methods

### Study design and participants

**Figure 1:**
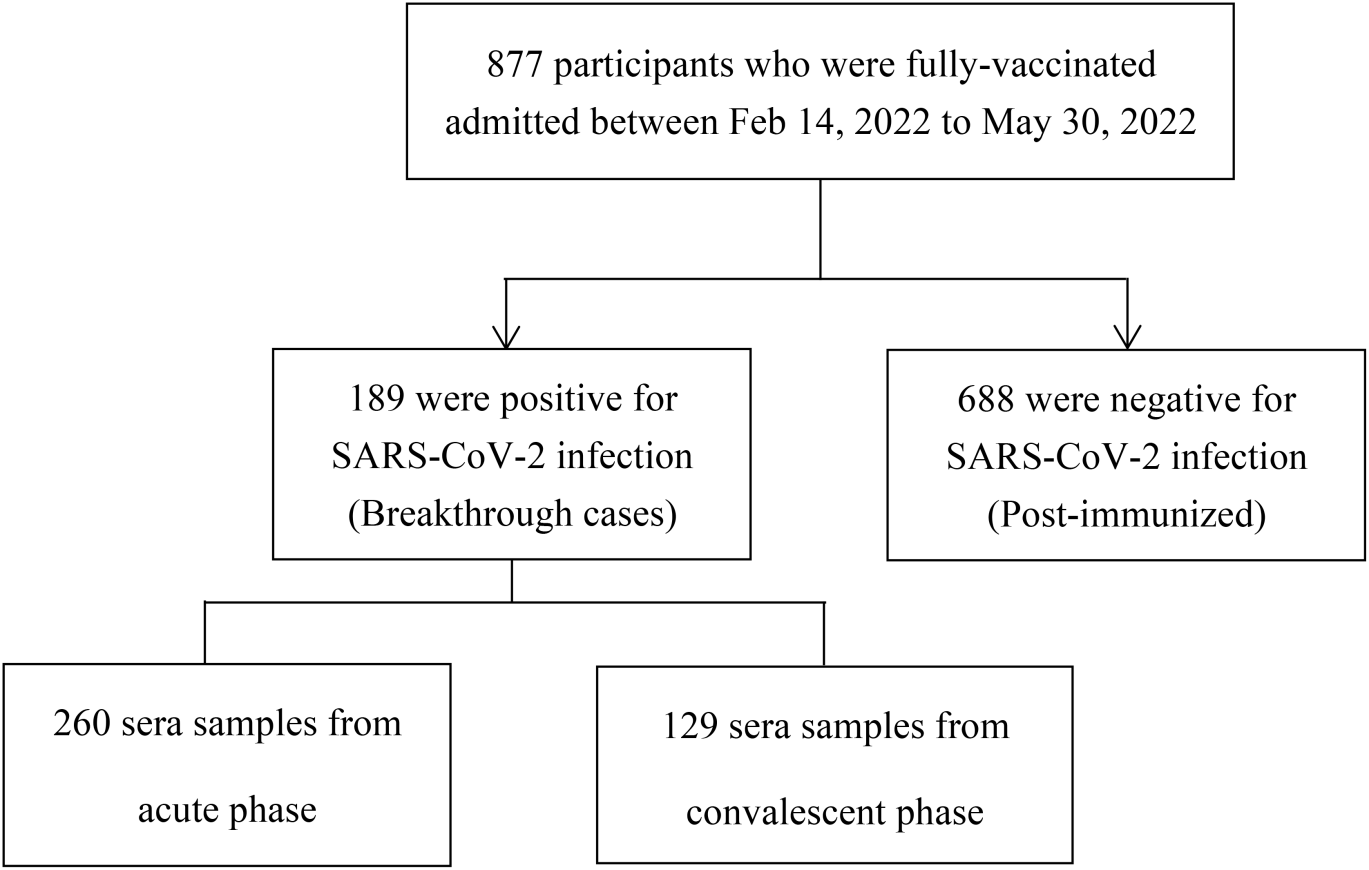
Flow chart of study enrollment.

This study included two groups: a breakthrough infection group, and a post-immunized group (**Figure 1**). The inclusion criteria of participants with breakthrough infections were: і. during the period from February 14, 2022, to May 30, 2022, in Jiangsu province, China, the individuals immunized with more than two doses of the inactive COVID-19 vaccine for over 2 weeks; ii. tested positive for nucleic acid of SARS-CoV-2. The inclusion criteria of post-immunized group were: і. From August 10, 2021, to August 20, 2021, individuals who had received at least two doses of inactive COVID-19 vaccine; ii. tested negative for nucleic acid of SARS-CoV-2. Exclusion criteria for both groups were including that: і.without clear immunization history; ii. no results of nucleic acid of SARS-CoV-2. Through whole-genome sequencing analysis (Illumina, CA, USA) (14), these participants were found to be infected with the Omicron BA.2 variant. In our study, we defined the booster dose as the third dose of COVID-19 vaccine. The acute phase was defined as the period of the first 2 weeks after disease onset. The convalescent phase was defined as the period after 2 weeks of disease onset.

From August 10, 2021, to August 20, 2021, a total of 688 individuals who had received at least two doses of COVID-19 vaccine were included as a post-immunized group to determine the NAbs characteristics among vaccinated individuals without breakthrough infection. All participants without SARS-CoV-2 infection were vaccinated with more than two doses of inactive COVID-19 vaccine, either BBIBP or CoronaVac.

### Surrogate virus neutralization test

Surrogate virus neutralization titers (magnetism particulate immunochemistry luminescence method, MCLIA) in the serum samples were measured *in vitro* using receptor-binding domain (RBD)-NAbs using the competitive chemiluminescence method performed with an automated Axceed 260 analyzer, as described in a previous study (15), and accompanying immunoassay test kits. The test kit consisted of reagent 0 (magnetic particle receptor angiotensin-converting enzyme 2 [ACE2] antigen), reagent 1 (alkaline phosphatase-labeled S protein RBD), calibrator 1, calibrator 2, and other necessary auxiliary reagents. During the test, reagent 0, reagent 1, and the serum samples were added to the reaction tubes. If the sample contained NAb, it would compete with the magnetic particle-labeled ACE2 antigen to bind to the S protein RBD. The free components were then rinsed away. The substrate solution was added, substrate solution luminescence was catalyzed by alkaline phosphatase, and the relative light units (RLU) of each sample tube was quantified. The RLU of the sample was negatively correlated with the concentration of SARS-CoV-2-NAb in the sample, allowing us to calculate the titer of the surrogate virus neutralization test (AU/mL) in human serum samples. According to the threshold specified by the manufacturer of magnetism particulate immunochemistry luminescence method (MCLIA), titers <2 AU/mL were considered negative, and titers ≥2 AU/mL were considered positive. Higher titers indicate higher levels of NAbs. NAb levels above 30 AU/mL were uniformly recorded as >30 AU/mL, and it was difficult to distinguish the difference between the levels of NAbs when both subjects had titers >30 AU/mL. We performed an experiment to determine the optimal dilution for samples with prime NAb titers >30 AU/mL. Firstly, sera from 50 Ancestral strain SARS-CoV-2 infected individuals were tested for NAbs by micro-neutralization test and the surrogate virus neutralization test (MCLIA). Secondly, sera from these 50 patients tested by MCLIA with > 30 AU/mL were diluted 5 times, 10 times, and 50 times, respectively. Finally, by using the NAbs titers of the micro-neutralization test as a reference, the correlation between the three diluted-times NAbs titers and the reference were analyzed. The correlation coefficient *r* are 0.815, 0.831, and 0.841 at 5-times, 10-times, and 50-times dilution, respectively, so the experiment was repeated in this study with samples diluted 50 times if the prime titer reading of the NAb was > 30 AU/mL (**Table S1**). Dilution of 100 times would increase the consumption of detection reagents and would further prolong the detection time, so our research group did not validate the dilution of 100 times. In summary, the final titers were kept when the prime values were between 0 and 30 AU/mL, and the final titers were the values of 50-fold dilution when the prime values >30AU/mL, so that the range of NAb values in this study was 0-1500 AU/mL. Titers <2 AU/mL were considered negative, and titers ≥2 AU/mL were considered positive. If the true value was between 30 and 100, for example, 50 AU/mL, the prime titer was > 30 AU/mL, then the sample would be diluted 50 times, and the final titer was around 50 AU/mL (1 AU/mL × 50).

### Statistical analysis

Statistical analysis was performed using SPSS 26.0 (IBM SPSS Statistics, Armonk, NY, USA) and GraphPad PRISM 9.3.1 (GraphPad Software, San Diego CA, USA). Continuous variables were reported as the mean ± standard deviation (SD) or median and interquartile range (IQR). Categorical variables were summarized as counts and percentages. NAb titers were non-parametric and therefore log(2)-transformed to normalize before analysis using Student’s unpaired t-test and one-way ANOVA test. The geometric mean titre (GMT) was calculated with 95% confidence intervals (CI). Univariate and multivariate linear regression models were used to identify factors associated with NAb levels among all participants (the breakthrough infection and post-immunized groups combined) and among the breakthrough infection group. We further conducted ROC curve analysis to evaluate the ability of NAb to identify breakthrough infected individuals in the vaccinated population. Two-sided *p* values <0.05 were considered statistically significant.

## Results

### Demographic baseline characteristics of the participants

From February 14, 2022, to May 30, 2022, 389 serum samples (acute phase: 260, convalescent phase: 129) were collected from 189 individuals with breakthrough infection, and from August 10, 2021, to August 20, 2021, 688 serum samples were collected from 688 fully vaccinated individuals without documentation of previous SARS-CoV-2 infection (**Figure 1**). Participants in the breakthrough infection and post-immunized groups had all been vaccinated with the BBIBP (inactivated COVID-19 vaccine manufactured by Beijing Institute of Biological Products Co., Ltd.), CoronaVac (inactivated COVID-19 vaccine manufactured by Sinovac Life Sciences Co., Ltd), or both the BBIBP and CoronaVac vaccines. According to *Diagnosis and Treatment Protocol for Novel Coronavirus Pneumonia* (16), the clinical spectrum of COVID-19 ranged from mild to critical. The participant demographic characteristics are shown in **Table 1**.

**Table 1:** Demographic baseline characteristics of the participants.

	Total participants (N=877)	Breakthrough cases (n=189)	Post-immunized group (n=688)	*p* value
Age (Mean ± SD)	47.7 ± 14.7	44.1 ± 13.9	48.7 ± 14.4	<0.001
Sex (n [%of total])				<0.001
Female	605(68.9%)	116(61.4%)	489(71.1%)	
Male	272(31.0%)	73(38.6%)	199(28.9%)	
Vaccines (n[%of total])				<0.001
BBIBP	280(31.9%)	61(32.3%)	219(31.8%)	
CoronaVac	527(60.1%)	118(62.4%)	409(59.4%)	
BBIBP&CoronaVac	70(7.9%)	10(5.3%)	60(8.7%)	
Booster vaccination (n [%of total])				<0.001
Yes	95(10.9%)	92(48.7%)	3(0.4%)	
No	781(89.1%)	97(51.3%)	684(99.6%)	
Time from vaccination to symptom onset (days [IQR])				—
Two doses	215(202-259)	215(202-259)	—	
Booster dose	63(28-93)	63(28-93)	—	
Clinical classification (n [%of total])				—
Mild	41(4.6%)	41(21.9%)	—	
Moderate	52(5.9%)	52(27.5%)	—	
Asymptomatic	96(10.9%)	96(50.6%)	—	

### NAb levels in the breakthrough infection and vaccination-only groups

**Figure 2:**
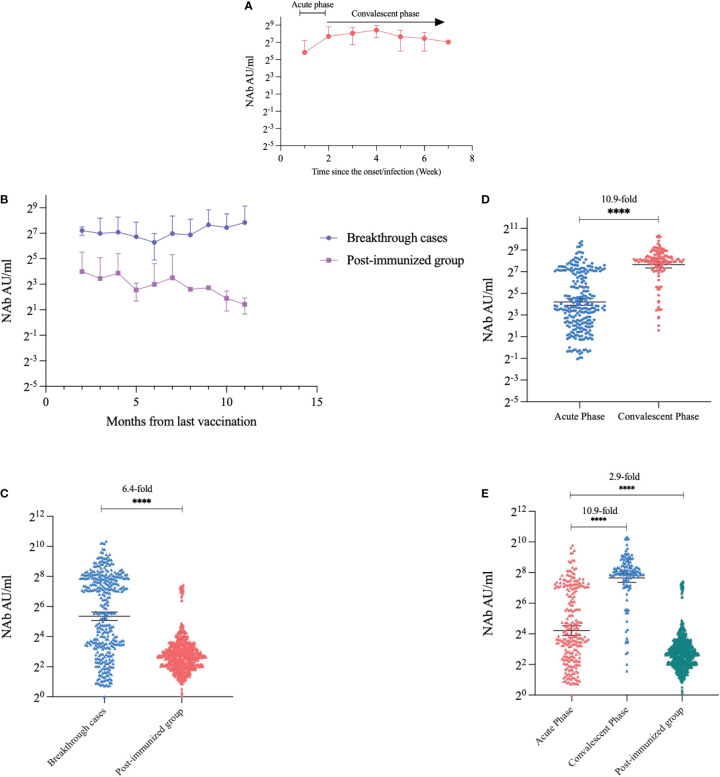
Kinetics and quantitative comparison of NAb levels over time. **(A)** Trends in NAb after the onset of breakthrough cases. **(B)** Trends in NAbs in breakthrough cases and post-immunized groups with time since the last vaccination. **(C)** Comparison of NAbs quantification in breakthrough cases and post-immunized group. **(D)** Comparison of NAbs in the acute phase and convalescent phase of breakthrough cases. **(E)** Comparison of NAbs in the acute phase, convalescent phase, and post-immunized group. Dark horizontal lines for each group denote sample medians, and the error bars and dotted lines indicate the interquartile range. ****P < 0.0001.

The kinetics of NAbs in participants with breakthrough infection showed that NAb titers started to increase in the second week after symptom onset, reached a small peak in the fourth week, and continued to slightly decrease thereafter (**Figure 2A**). We compared the NAb levels between the two groups at the same post-vaccination time point, taking into account that NAbs wane after vaccination. We found that the breakthrough infection group had consistently higher NAb values than the post-immunized group according to time since the last vaccination (*p*<0.0001) (**Figure 2B**). Nab levels of the breakthrough infection group were 6.4-fold higher than those of the post-immunized group (GMT: 40.72 AU/mL and 6.38 AU/mL, respectively, *p*<0.0001) (**Figure 2C**). We compared NAb levels between the acute and convalescent phases in participants with breakthrough infection. The NAbs of the convalescent phase were 10.9-fold higher than those of the acute phase (GMT: 200.48 AU/mL and 18.46 AU/mL, respectively, *p*<0.0001) (**Figures 2D, E**), violin plots are shown in **Supplementary Material Figure S1**.

**Figure 3:**
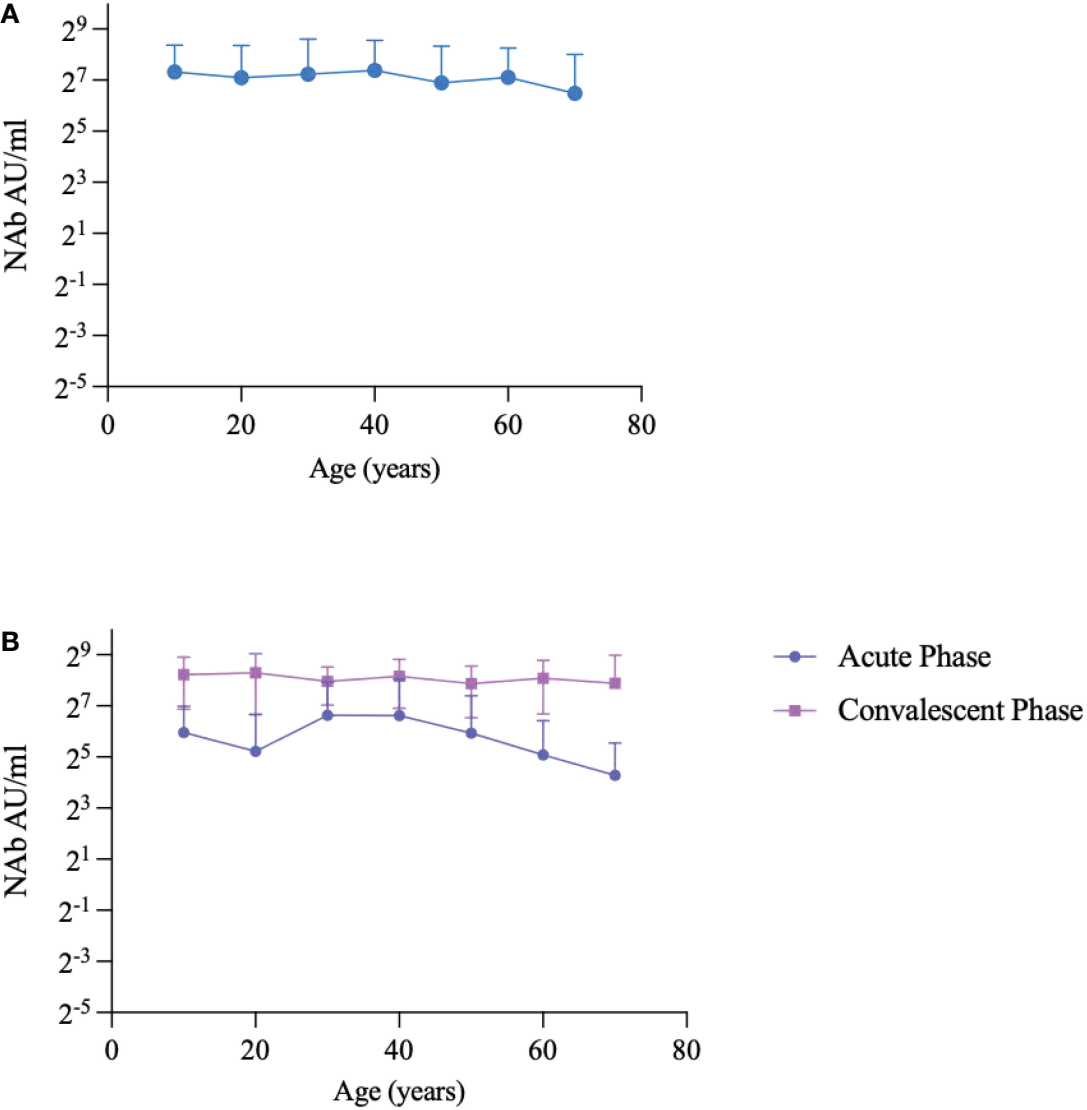
Changes in NAbs with age. **(A)** Changes in NAbs with age in breakthrough cases. **(B)** Changes in NAbs with age during the acute phase and convalescent phase. Each circle and triangle represent the titer for a serum sample. Note: Breakthrough cases were included from February 14, 2022, to May 30, 2022, post-immunized groups were recruited from August 10, 2021, to August 20, 2021.

We further analyzed differences in NAb levels with age. The NAb levels in participants with breakthrough infection showed a slight downward trend with age (**Figure 3A**). In addition, the NAb levels in age groups over 60-year were higher in the convalescent phase than those in the acute phase (*p*<0.0001). In the acute phase, NAb levels peaked in the 30-year-old group and then steadily decreased with age. However, NAb levels in the convalescent phase did not vary significantly with age (**Figure 3B**).

### Impact of the time since SARS-CoV-2 vaccination to NAb measurement in participants with breakthrough infections

**Figure 4:**
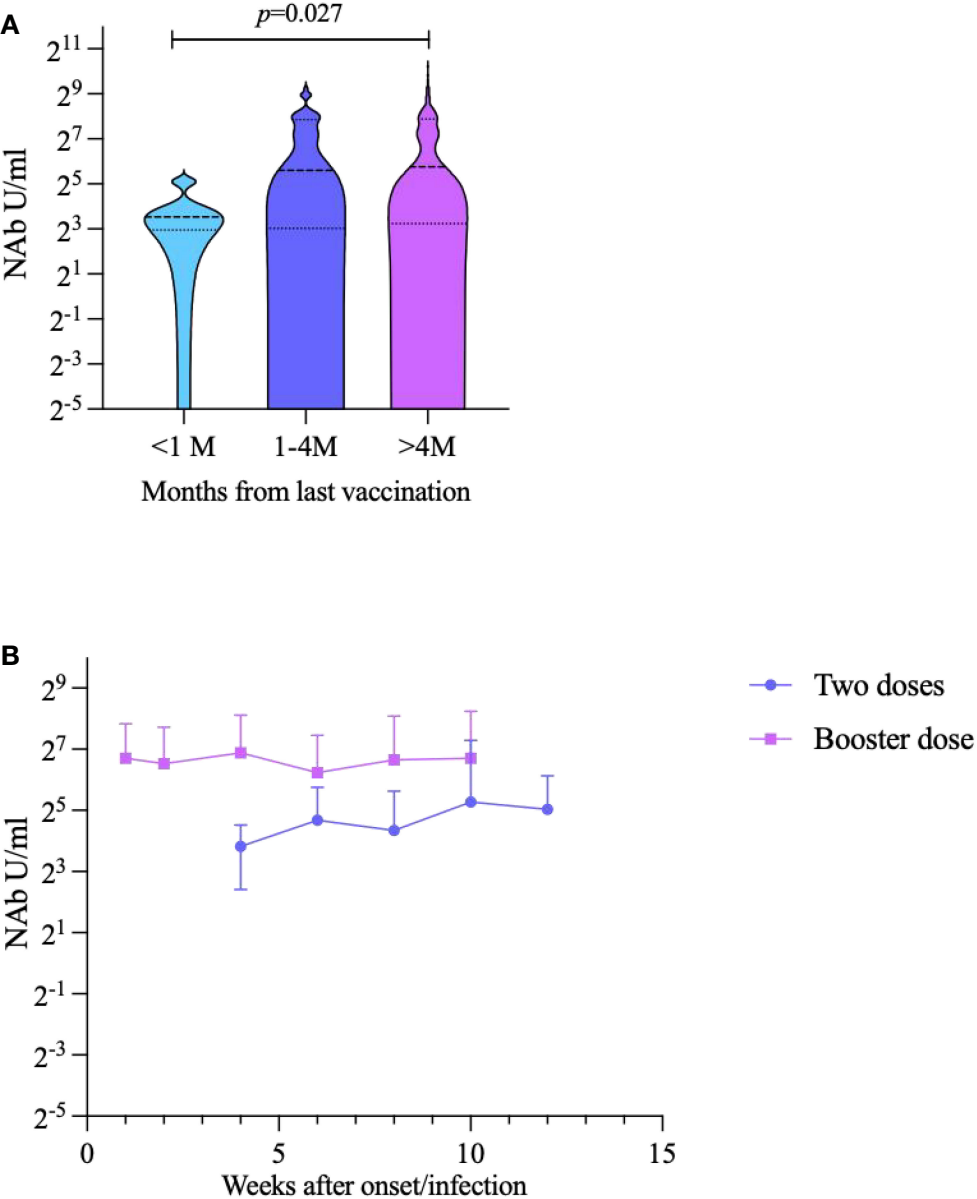
Impact of inactivates SARS-CoV-2 vaccination to NAbs against Omicron BA.2 variant in breakthrough cases. **(A)** Breakthrough case classification analysis according to the time since the last vaccination to sampling. **(B)** Trends in NAb over time for different vaccination doses.

We analyzed NAbs of breakthrough infection according to the time between the last vaccination to sampling. We divided the timing of vaccination into three groups: <1 month, 1–4 months, and >4 months. The results showed there was statistically significant difference of NAb levels between <1 month group and >4 months group (*p*=0.027) (**Figure 4A**). Subsequently, we analyzed the trend in NAb levels over time for different vaccination doses in participants with breakthrough infection. The NAb levels of participants who had received a booster dose were consistently higher than those of participants who had received only two doses of vaccine at every time point (53.16 AU/mL and 30.97 AU/mL, respectively; *p=*0.007) (**Figure 4B**).

### Linear regression analysis of the factors influencing NAb levels

Univariate regression and multivariate linear regression analyses were conducted to quantify the contributions of the variables to NAbs titers in all participants combined, and the breakthrough infection group. After adjusting for demographic variables, we found that the factors influencing NAb levels in all participants were breakthrough infection, age, and booster vaccination (**Table 2**). Specifically, for each additional breakthrough infection relative to vaccination alone, the NAb levels increased by log(2)-transformed 2.3 units. Using the <20 years age group as the reference group, the 30-40/40-50/50-60-years age group were significantly negatively associated with NAbs (β ± SD: −0.684 ± 0.314, −0.592 ± 0.294, −0.880 ± 0.305, for the 30–40, 40–50, 50–60 years age groups, respectively). Participants who had received a booster dose of vaccine had a mean NAb level that was log(2)-transformed 0.8 units higher than that of those who had not received a booster dose.

**Table 2:** Linear regression of the factors influencing NAb levels in the whole participants.

	Univariate regression analysis				Multi-factor regression analysis			
	Unstandardized Coefficient		Standardized Coefficient	*p* value	Unstandardized Coefficient		Standardized Coefficient	*p* value
All participants	B	Standard error	β		B	Standard error	β	
Status of infection								
Breakthrough cases	2.690	0.128	0.541	<0.0001	2.264	0.176	0.455	<0.001
Post-immunized group	Reference				Reference			
Sex								
Male	0.764	0.147	0.157	<0.0001	-0.056	0.133	-0.011	0.674
Female	Reference				Reference			
Age (years)								
>70	-2.532	0.409	-0.263	<0.0001	-0.461	0.371	-0.048	0.214
60-70	-2.431	0.351	-0.370	<0.0001	-0.551	0.318	-0.084	0.083
50-60	-2.028	0.346	-0.324	<0.0001	-0.880	0.305	-0.140	0.004
40-50	-1.754	0.334	-0.318	<0.0001	-0.592	0.294	-0.107	0.044
30-40	-2.653	0.345	-0.427	<0.0001	-0.684	0.314	-0.110	0.030
20-30	-2.108	0.369	-0.278	<0.0001	-0.547	0.328	-0.072	0.096
<20	Reference				Reference			
Booster vaccination								
Yes	2.552	0.171	0.414	<0.0001	0.775	0.202	.126	<0.0001
No	Reference				Reference			

Among participants with breakthrough infection and a clear history of infection, we further used linear regression to analyze the correlation between the time since infection and the quantitative value of NAbs. In the breakthrough infection group, the time since infection, booster vaccination and the time from the last vaccination to symptom onset were significantly associated with log(2)-transformed NAb levels; however, sex, age, and clinical status were not (**Table 3**). Specifically, the NAb levels increased by log(2)-transformed 2.4, 3.4, 4.5, and 2.9 units in the second, third, fourth, and 5 weeks or more after infection, respectively, compared with the first week after infection. Booster vaccination was associated with an increase of log(2)-transformed 0.7 units of NAb relative to those who did not receive the booster dose. The NAb levels increased by log(2)-transformed 4.7 and 4.9 units in the 1-4 months and over 4 months, respectively, compared with the first month after last vaccination. ROC curve analysis showed that ROC area was largest at 0.728 when the cut-off value of log(2)-transformed NAb was 6. The results in details are shown in **Supplementary Material Table S2**.

**Table 3:** Linear regression of the factors influencing NAb levels in the breakthrough cases.

	Univariate regression analysis				Multi-factor regression analysis			
	Unstandardized Coefficient		Standardized Coefficient	*p* value	Unstandardized Coefficient		Standardized Coefficient	*p* value
Breakthrough cases	B	Standard error	β	B	Standard error	β		
Weeks after infection								
First week	Reference				Reference			
Second week	2.477	0.283	0.411	<0.0001	2.362	0.283	0.391	<0.001
Third week	3.404	0.444	0.352	<0.0001	3.425	0.442	0.354	<0.001
Fourth week	4.423	1.023	0.192	<0.0001	4.465	1.012	0.194	<0.001
Over five weeks	3.059	0.478	0.292	<0.0001	2.923	0.477	0.297	<0.001
Sex								
Male	0.233	0.296	0.040	0.432	0.012	0.262	0.002	0.964
Female	Reference				Reference			
Age (years)								
>70	-1.377	0.978	-0.077	0.160	-1.037	0.839	-0.058	0.217
60-70	-0.739	0.611	-0.129	0.227	-0.605	0.526	-0.062	0.251
50-60	-.885	0.488	-0.132	0.071	-0.690	0.425	-0.103	0.105
40-50	-0.274	0.463	-0.045	0.554	-0.157	0.402	-0.026	0.697
30-40	-0.820	0.622	-0.082	0.188	-0.173	0.543	-0.017	0.750
20-30	-1.024	0.597	-0.108	0.087	-0.921	0.513	-0.097	0.073
<20	Reference				Reference			
Booster vaccination								
Yes	0.779	0.286	0.137	0.007	0.732	0.253	0.129	0.004
No	Reference				Reference			
Clinical status								
Asymptomatic	-0.157	0.349	-0.028	0.652	—			
Mild	0.103	0.416	0.015	0.804	—			
Moderate	Reference							
Months since last vaccination								
>4 month	6.064	1.997	0.715	0.003	4.864	1.736	0.573	0.005
1-4 month	5.778	2.033	0.669	0.005	4.691	1.769	0.543	0.008
< 1 month	Reference				Reference			

## Discussion

This study with 877 participants and 1077 serum samples showed that NAb levels of participants with breakthrough SARS-CoV-2 Omicron subvariant BA.2 infections were six-fold greater than those of the post-immunized group. The participants with breakthrough infection had enhanced serum-neutralizing responses compared with participants who received the COVID-19 vaccine only, considering the attenuation of neutralizing antibodies induced by inactivated vaccination over time, in agreement with a previous study (17). We found that the time since infection was the main factor influencing the NAb titer in participants with breakthrough infection, which showed that NAbs in the convalescent phase were almost 11-fold higher than those in the acute phase, which is consistent with a study by Oliveira and colleagues (18).

SARS-CoV-2 breakthrough infections occur when an individual has been fully vaccinated and, subsequently is infected with the most recent circulating SARS-CoV-2 variant (19, 20). Breakthrough infections induce potent NAb responses. A previous study reported that serological titers of NAb rapidly increased and were maintained after antigen re-exposure in vaccinated individuals owing to recalled immune memory (21). When vaccinated individuals are infected with SARS-CoV-2, similar to the initial infection, as the virus continues to replicate, more NAbs are produced in the human body to neutralize virus particles, as evidenced by higher levels of NAb during the convalescent phase than during the acute phase, and by the gradual increase in quantitative levels of NAbs and a plateau at a higher level within one month after infection (22, 23).

The impact of the NAbs titer dynamics of breakthrough infections involved several factors, including the individuals’ inherent response to the vaccine regime, virulence, and the duration of a protective level of NAbs. In addition, pre-existing conditions vary from one individual to the next, contributing to breakthrough infections, including but not limited to immune suppression, age, genetics, and a variety of other underlying comorbidities. According to our results, NAbs decreased with age, mainly in the acute phase, but were not significantly associated with age in the convalescent phase. Patients of different ages during the convalescent phase have had sufficient time to develop NAbs against SARS-CoV-2; therefore, age is not a major influencing factor during this period. Studies have shown that there is no correlation between NAbs and COVID-19 disease severity (24). Months from the last vaccination to symptom onset was significantly and positively associated with NAb levels in the regression analysis. In breakthrough cases, it appears that those who received their vaccinations earlier than those who received them later had higher levels of NAbs, which is consistent with our previous study (25).

Regression analysis for the two cohorts shows that breakthrough infection leads to a further increase in NAb levels in vaccinated individuals. These data lend support for tracing or identifying previously infected individuals. SARS-CoV-2 nucleic acid remains in the body for approximately 1~2 weeks, but NAbs tend to remain in the body for a longer period. When throat swab real-time PCR results were negative, we used the NAb levels to distinguish individuals with breakthrough infection from vaccinated individuals without infection. In regression analysis for breakthrough infection group, as NAb levels rise after 2 weeks of infection, it is difficult to accurately determine the precise time since infection based on the specific NAb value, but NAb values can be used to estimate the relative time range. The results of the ROC curve analysis also indicate that NAb levels better identify breakthrough infected individuals from the vaccinated population. However, the value of quantitative NAb values in traceability will be diminished during the later phase of pandemic.

This study has several limitations. First, this study used the magnetism particulate immunochemistry luminescence method instead of authentic microneutralization. Previous research by our group has shown a good correlation between the surrogate virus neutralization test and authentic microneutralization (26). In participants with high NAb levels, we determined the appropriate dilution ratio by comparing the correlation between the results of the surrogate virus neutralization test and those of authentic microneutralization test at different dilution ratios. Second, the antigen used in this assay was the wild strain of SARS-CoV-2, which has some differences from the Omicron variant in amino acids with the RBD region of the S protein, therefore the results could be just used for reference and speculation.

## Conclusion

Overall, our study showed that NAb titers participants with breakthrough infection were six-fold higher than those of the post-immunized group, and that the NAb levels during the convalescent phase were almost eleven-fold higher than those during the acute phase. Breakthrough infection can lead to a further increase in the NAb levels of the immunized population. When throat swab nucleic acid real-time PCR results are negative, NAb levels can be used to identify individuals with breakthrough infection.

## Data availability statement

The original contributions presented in the study are included in the article/**Supplementary Material**. Further inquiries can be directed to the corresponding authors.

## Ethics statement

The studies involving human participants were reviewed and approved by the Institutional Review Board of Jiangsu Provincial Center for Disease Control and Prevention (JSJK2021-B013-01). Written informed consent to participate in this study was provided by the participants’ legal guardian/next of kin. The patients/participants provided their written informed consent to participate in this study.

## Author contributions

AS, formal analysis, data curation, laboratory detection, statistical analysis, plotting, writing original draft preparation, writing review, and editing. CL, CC, CD, HT, XK, LZ, and JP, field survey and laboratory detection. SZ, data curation. FZ and CB, supervision. HJ, and YW, investigation and supervision. LGZ, supervision, original draft preparation, writing review, and editing. All authors contributed to the article and approved the submitted version.

## References

[1] CDC COVID-19 Vaccine Breakthrough Case Investigations Team. COVID-19 vaccine breakthrough infections reported to CDC - united states, January 1–April 30, 2021. MMWR Morb Mortal Wkly Rep (2021) 70(21):792–3. doi: 10.15585/mmwr.mm7021e3 PMC8158893 34043615

[2] Centers for Disease Control and Prevention. When you’ve been fully vaccinated. Available at: https://www.cdc.gov/coronavirus/2019-ncov/vaccines/fully-vaccinated.html (Accessed 8 November 2021).

[3] Di Fusco M Moran MM Cane A Curcio D Khan F Malhotra D. Evaluation of COVID-19 vaccine breakthrough infections among immunocompromised patients fully vaccinated with BNT162b2. J Med Econ (2021) 24(1):1248–60. doi: 10.1080/13696998.2021.2002063 34844493

[4] Ai J Zhang H Zhang Y Lin K Zhang Y Wu J. Omicron variant showed lower neutralizing sensitivity than other SARS-CoV-2 variants to immune sera elicited by vaccines after boost. Emerg Microbes Infect (2022) 11(1):337–43. doi: 10.1080/22221751.2021.2022440 PMC8788341 34935594

[5] Viana R Moyo S Amoako DG Tegally H Scheepers C Althaus CL. Rapid epidemic expansion of the SARS-CoV-2 omicron variant in southern Africa. Nature (2022) 603:679–86. doi: 10.1038/s41586-022-04411-y PMC8942855 35042229

[6] Cameroni E Bowen JE Rosen LE Saliba C Zepeda SK Culap K. Broadly neutralizing antibodies overcome SARS- CoV-2 omicron antigenic shift. Nature (2022) 602:664–70. doi: 10.1038/s41586-021-04386-2 PMC9531318 35016195

[7] Cao Y Wang J Jian F Xiao T Song W Yisimayi A. Omicron escapes the majority of existing SARS-CoV-2 neutralizing antibodies. Nature (2022) 602:657–63. doi: 10.1038/s41586-021-04385-3 PMC8866119 35016194

[8] Gruell H Vanshylla K Tober-Lau P Hillus D Schommers P Lehmann C. mRNA booster immunization elicits potent neutralizing serum activity against the SARS-CoV-2 omicron variant. Nat Med (2022) 28:477–80. doi: 10.1038/s41591-021-01676-0 PMC8767537 35046572

[9] Jiang S Zhang X Yang Y Hotez PJ Du L. Neutralizing antibodies for the treatment of COVID-19. Nat Biomed Eng (2020) 4:1134–9. doi: 10.1038/s41551-020-00660-2 PMC7891858 33293725

[10] Bhattacharya M Chatterjee S Mallik B Sharma AR Chakraborty C. Therapeutic role of neutralizing antibody for the treatment against SARS-CoV-2 and its emerging variants: A clinical and pre-clinical perspective. Vaccines (Basel) (2022) 10(10):1612. doi: 10.3390/vaccines10101612 36298477 PMC9606861

[11] Garcia-Beltran WF Lam EC Astudillo MG Yang D Miller TE Feldman J. COVID-19 neutralizing antibodies predict disease severity and survival. Cell (2021) 184(2):476–488.e11. doi: 10.1016/j.cell.2020.12.015 PMC7837114 33412089

[12] Zhu FC Meng FY Li JX Li XL Mao QY Tao H. Efficacy, safety, and immunology of an inactivated alum-adjuvant enterovirus 71 vaccine in children in China: A multicentre, randomised, double-blind, placebo-controlled, phase 3 trial. Lancet (2013) 381(9882):2024–32. doi: 10.1016/S0140-6736(13)61049-1 23726161

[13] Huang KA Zhou D Fry EE Kotecha A Huang PN Yang SL. Structural and functional analysis of protective antibodies targeting the threefold plateau of enterovirus 71. Nat Commun (2020) 11(1):5253. doi: 10.1038/s41467-020-19013-3 33067459 PMC7567869

[14] Wang S Zou X Li Z Fu J Fan H Yu H. Analysis of clinical characteristics and virus strains variation of patients infected with SARS-CoV-2 in jiangsu province-a retrospective study. Front Public Health (2021) 9:791600. doi: 10.3389/fpubh.2021.791600 35004593 PMC8739897

[15] Cheng ZJ Huang H Zheng P Xue M Ma J Zhan Z. Humoral immune response of BBIBP COVID-19 vaccination before and after the booster immunization. Allergy (2022) 7:10. doi: 10.1111/all.15271 PMC9111230 35255171

[16] Diagnosis and treatment protocol for novel coronavirus pneumonia (Trial version 7). Chin Med J (Engl) (2020) 133(9):1087–95. doi: 10.1097/CM9.0000000000000819 PMC7213636 32358325

[17] Walls AC Sprouse KR Bowen JE Joshi A Franko N Navarro MJ. SARS-CoV-2 breakthrough infections elicit potent, broad, and durable neutralizing antibody responses. Cell (2022) 185(5):872–880.e3. doi: 10.1016/j.cell.2022.01.011 35123650 PMC8769922

[18] Oliveira CF Neto WFF Silva CPD Ribeiro ACS Martins LC Sousa AW. Absence of anti-RBD antibodies in SARS-CoV-2 infected or naive individuals prior to vaccination with CoronaVac leads to short protection of only four months duration. Vaccines (Basel) (2022) 10(5):690. doi: 10.3390/vaccines10050690 35632447 PMC9147084

[19] Gupta RK Topol EJ. COVID-19 vaccine breakthrough infections. Science (2021) 6575:1561–2. doi: 10.1126/science.abl8487 34941414

[20] Feikin DR Higdon MM Abu-Raddad LJ Andrews N Araos R Goldberg Y. Duration of effectiveness of vaccines against SARS-CoV-2 infection and COVID-19 disease: Results of a systematic review and meta-regression. Lancet (2022) 10328:924–44. doi: 10.1016/S0140-6736(22)00152-0 PMC8863502 35202601

[21] Koutsakos M Lee WS Reynaldi A Tan HX Gare G Kinsella P. The magnitude and timing of recalled immunity after breakthrough infection is shaped by SARS-CoV-2 variants. Immunity (2022) 55(7):1316–1326.e4. doi: 10.1016/j.immuni.2022.05.018 35690062 PMC9135796

[22] Miyamoto S Arashiro T Adachi Y Moriyama S Kinoshita H Kanno T. Vaccination-infection interval determines cross-neutralization potency to SARS-CoV-2 omicron after breakthrough infection by other variants. Med (NY) (2022) 3(4):249–261.e4. doi: 10.1016/j.medj.2022.02.006 PMC8894731 35261995

[23] Lyke KE Atmar RL Islas CD Posavad CM Szydlo D Paul Chourdhury R. Rapid decline in vaccine-boosted neutralizing antibodies against SARS-CoV-2 omicron variant. Cell Rep Med (2022) 3(7):100679. doi: 10.1016/j.xcrm.2022.100679 35798000 PMC9212999

[24] Robbiani DF Gaebler C Muecksch F Lorenzi JCC Wang Z Cho A. Convergent antibody responses to SARS-CoV-2 in convalescent individuals. Nature (2020) 584:437–42. doi: 10.1038/s41586-020-2456-9 PMC7442695 32555388

[25] Zhu L Mao N Yi C Simayi A Feng J Feng Y. Impact of vaccination on kinetics of neutralizing antibodies against SARS-CoV-2 by serum live neutralization test based on a prospective cohort. Emerg Microbes Infect (2022) 14:1–73. doi: 10.1080/22221751.2022.2146535 PMC9858416 36373485

[26] Kong X Zhang S Li Z Yu H Zou X Tian H. Evaluation of high-throughput methods for the detection of neutralizing antibodies in serum samples from individuals infected with 2019-nCoV based on a microneutralization test. Chin J Exp Clin Virol (2022) 36(03):306–10. doi: 10.3760/cma.j.cn112866-20211109-00198

