# Supplementary material for: Kinetics of SARS-CoV-2 neutralizing antibodies in Omicron breakthrough cases with inactivated vaccination: Role in inferring the history and duration of infection
Source: Front Immunol. 2023 Jan 24;14:1083523. doi: 10.3389/fimmu.2023.1083523 (PMC9902649; doi:10.3389/fimmu.2023.1083523)
Supplement: Supplementary file 1 [file DataSheet_1.zip › Data Sheet1/Supplementary material Fig S1.docx]

**A**


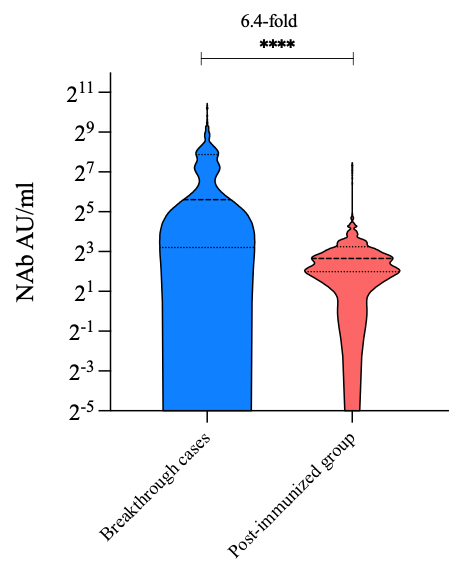


**B**


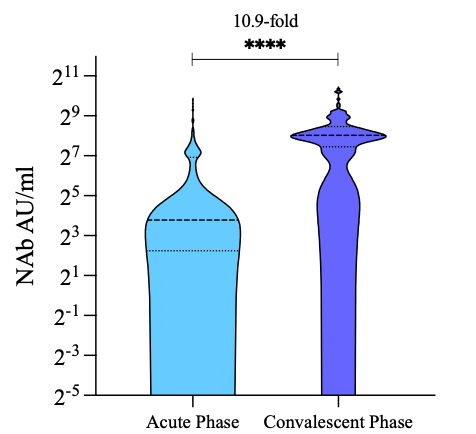


**C**


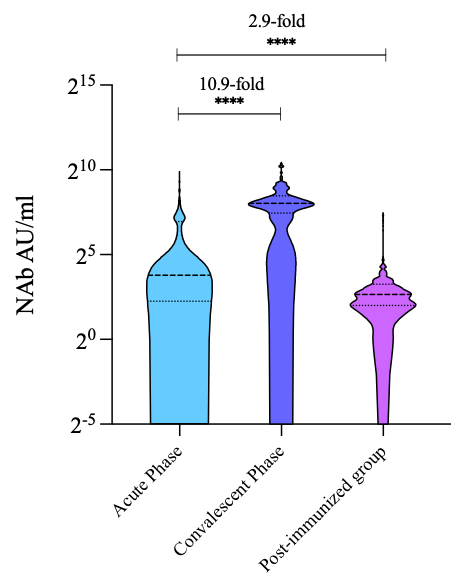


**Fig.S1 Violin plots of quantitative comparison of NAb levels over time. (A)** Comparison of NAbs quantification in breakthrough cases and post-immunized group. **(B)** Comparison of NAbs in the acute phase and convalescent phase of breakthrough cases. **(C)** Comparison of NAbs in the acute phase, convalescent phase, and post-immunized group. *****P* < 0.0001.
