# Supplementary material for: Kinetics of SARS-CoV-2 neutralizing antibodies in Omicron breakthrough cases with inactivated vaccination: Role in inferring the history and duration of infection
Source: Front Immunol. 2023 Jan 24;14:1083523. doi: 10.3389/fimmu.2023.1083523 (PMC9902649; doi:10.3389/fimmu.2023.1083523)
Supplement: Supplementary file 2 [file Table_1.docx]

**Supplementary material**

First, sera from 50 randomly selected SARS-CoV-2 infected individuals of the Ancestral strain were tested for NAbs by live virus neutralization assay and chemiluminescence. Sera with antibody results greater than 30 were diluted 5 times, 10 times, and 50 times, respectively. By using the antibody results of the micro-neutralization test as a reference, the correlation between the different diluted NAb results was done and the diluted NAb results were analyzed, and the best dilution concentration of the serum was the one with the largest correlation coefficient. The results are shown in Table S1 and the phase relationship increased from 0.815 to 0.841 at 50-fold dilution, i.e., the correlation was strong, so the experiment was repeated in this study with samples diluted 50 times if the final titer reading of the NAb was greater than 30.

**Table S1. Correlation analysis of different dilution concentrations NAb results.** Spearman’s rank coefficient (r) was used to assess the strength of correlation and to determine the *p*-value for significance.

|  | Chemiluminescence dilution times | Correlation coefficient r | *p* value |
| --- | --- | --- | --- |
| Live virus neutralization assay | Diluted 50 times | 0.841 | <0.01 |
|  | Diluted 10 times | 0.831 | <0.01 |
|  | Diluted 5 times | 0.815 | <0.01 |
