# Supplementary material for: Kinetics of SARS-CoV-2 neutralizing antibodies in Omicron breakthrough cases with inactivated vaccination: Role in inferring the history and duration of infection
Source: Front Immunol. 2023 Jan 24;14:1083523. doi: 10.3389/fimmu.2023.1083523 (PMC9902649; doi:10.3389/fimmu.2023.1083523)
Supplement: Supplementary file 3 [file Table_2.docx]

**Table S2.** Ability to distinguish the breakthrough cases from the post-immunized population by different Cutoff values for the NAb.

| Cut-off Value of log(2)-transformed NAb | Sensitivity（%） | Specificity（%） | Positive predictive value （%） | Negative predictive value （%） | ROC Area |
| --- | --- | --- | --- | --- | --- |
| 6 | 48.72 | 96.78 | 89.62 | 76.80 | 0.728 |
| 6.5 | 46.41 | 97.08 | 90.05 | 76.06 | 0.717 |
| 7 | 43.85 | 98.10 | 92.93 | 75.39 | 0.710 |
